# Supplementary material for: Are carbon emissions trading and green financial instruments synergistic? -Comprehensive quantitative research based on content analysis
Source: PLoS One. 2024 Mar 7;19(3):e0298601. doi: 10.1371/journal.pone.0298601 (PMC10919601; doi:10.1371/journal.pone.0298601)
Supplement: S1 Data — (ZIP) [file pone.0298601.s001.zip › Data/地级市Z_F工作报告.zip!/╣π╢1⁄2/╔╟═╖╩╨/└┤╫╘╣1⁄2╓┌║┼í╛┬φ┐╦╩2╛▌═°í┐_2014.docx]

各位代表：

　　我代表汕头市人民政府，向大会报告工作，请予审议，并请各位政协委员和其他列席人员提出意见。

　　一、2013年工作回顾

　　过去一年，面对复杂严峻的国内外经济形势，面对“8·17”重大洪涝灾害和强台风“天兔”的袭击，全市人民在省委、省政府和市委的正确领导下，团结一心，奋力拼搏，一手抓经济社会发展，一手抓救灾复产，全市经济社会发展保持稳中有快的良好态势。全市生产总值1565.9亿元，增长10%，高于全国、全省平均水平，比上一年提高0.5个百分点。来源于我市的财政收入277.92亿元，其中公共财政预算收入突破百亿元大关，达112.11亿元，增长16.4%。固定资产投资额784.67亿元，增长28.2%，连续三年高速增长且增速位居全省前列；全年新登记各类企业3671户，其中注册资本超过1000万元的134户，新一轮加快发展、跨越发展的基础正在不断夯实。一年来，各项工作取得了实质性成效。

　　一是交通基础设施建设大会战捷报频传。集中资源和力量，上下联动，各方配合，千方百计破解资金瓶颈和征地拆迁难题，掀起一场轰轰烈烈的交通大会战。汕头大地上四条高速公路全面开工建设，汕揭梅高速月浦至泰山立交段建成通车，泰山立交至汕汾高速段建设进展顺利，汕湛、潮惠、揭惠高速汕头段开工建设，潮汕环线高速公路按省市共建模式启动勘察设计和项目投资人招标，汕头海湾隧道完成勘察设计招标。厦深铁路开通运行，潮阳站站房主体工程及进出站路建成投入使用。厦深联络线经多方争取，初步设计方案获省政府和中国铁路总公司批复同意实施，疏港铁路、广梅汕铁路扩能改造项目前期工作顺利推进，粤东城际和市域轨道交通项目完成初步规划编制。干线公路升级改造步伐加快，国道206线大学路改造完成一期工程，启动二期工程建设，国道324线外砂段完工，潮阳、潮南段路面改造及峡山段改线工程开工建设；省道234线、235司神线、231凤湾线以及省道237和337线和惠公路二期工程、磊广路、河浦大道等改造工程开工建设；南澳大桥主桥主塔封顶。广澳港区防波堤、海门港区华能煤炭中转基地加快建设。

　　二是大特区城市规划建设全面铺开。按照建设创新型经济特区、东南沿海现代化港口城市、粤东地区中心城市的发展定位，推进新一轮城市总体规划修编，以规划建设海湾新区为重点，推动形成“一湾两岸”城市核心圈、“一核多组团”大特区城市格局。海湾新区完成总体规划编制，在核心区36平方公里创办华侨经济文化合作试验区，起步区12平方公里完成控制性详细规划和城市设计，规划展示馆建成对外开放。海湾新区“六城一区”全面启动建设，东海岸新城累计投资近70亿元，基本完成围海造地工程，形成陆域面积20平方公里、海堤24公里，新津片区首期约480亩土地挂牌出让，中山路东延段、滨江大道、新津河大桥、外砂河大桥等市政设施启动建设；珠港新城完成控制性详细规划，启动土地一级开发，中国卫星航天技术展示厅建成投入使用，国瑞会展酒店动工；濠江新城完成南滨片区项目工可和市政规划编制，启动土地一级开发和中轴线景观工程、潮汕历史文化博览中心建设；中国锆城列入省第二批战略性新兴产业基地，核用条带项目、2700吨复合氧化锆项目建成投产；澄海六合新城启动围海造地论证和规划编制；粤东物流新城完成土规调整、南片区和泰山路征地，首批项目用地挂牌出让，泰山路北延工程启动建设；南澳被认定为省滨海旅游产业园，完成龙门湾滨海新城区域性用海规划编制、长山尾围地项目招投标前期工作。积极推进西部生态智慧新城、潮阳东部新城、潮南行政新区、厦深高铁潮阳站（谷饶）物流新城等战略合作开发。加快城市更新步伐，启动建设小公园开埠历史文化街区、潮人码头，改造大华路、外马路、西堤路、海河路等共16.45万平方米破旧道路，启动和完成改造升级潮阳、潮南、澄海、南澳城区一大批市政道路，城乡面貌大为改善更新。加大环境保护力度，严把建设项目环保准入关，加快一批环保基础设施建设，加强印染、电镀等重点行业和贵屿、练江等重点区域的污染治理，节能减排指标可望完成年度目标。

　　三是现代产业加快发展。全面实施四大产业计划，扎实推进“五个100工程”，产业结构不断优化，特别是现代服务业投资延续高增长态势，达到25.1%。继续开展重点项目集中开工季活动，集中开工项目180个，总投资额729亿元，其中现代产业项目投资额146亿元，占比超过20%。宝奥物流城加快建设正大配送中心、货物超市和国际玩具城，完成一期招商。“数据特区”正加紧与国家互联网权威管理机构和行业龙头企业开展战略合作，共同发展国际海缆登陆站经济。与中国卫星、国药集团和香港中航科技等合作的一批产业项目有序推进。星湖城商业综合体、龙光喜来登酒店投入运营，苏宁电器广场、百脑汇数码广场、正大广场、华润万家、长平新一城、猛狮国际广场、潮南巴比伦商贸城等一批商业综合体项目加快推进。一批电子商务产业园和骨干企业迅猛发展，苏宁易购粤东地区电子商务运营中心开工建设，全市应用电子商务的市场主体突破10万家，交易规模超400亿元，增长超50%，增速居全国前列。金融业加快发展，新增东亚银行、恒生银行等8家金融机构，汕头金融超市建成运营。金融机构本外币存款余额2530.16亿元，贷款余额971.93亿元，分别增长10.7%、19.7%。农业生产加速向园艺化、设施化、标准化转型，特色效益农业成为农业家庭经营支柱，全市累计农业设施化生产面积达到10万亩以上。鼓励企业开展技术创新，新组建25家省级、22家市级工程技术研究开发中心，1家省重点实验室；新增专利授权6833件，其中发明专利211件，增长14.7%。制定出台促进工业园区发展的指导意见及配套文件，推动产业集聚发展。

　　四是开放合作不断深化。成功举办第五届粤东侨博会，吸引来自20个国家和地区的50个社团代表、500名海外嘉宾参加，签订粤东五市加快推进交通基础设施建设协作协议、合作创办广东以色列理工学院框架协议，签约项目185个，投资额1948亿元。大力推进电子口岸建设，启动关检合作“一次申报、一次查验、一次放行”通关模式试点，通关环境进一步优化，全年进出口总额92.34亿美元，增长4.9%，其中出口总额66.02亿美元，增长7.1%。帮助企业开拓国际市场，组织企业参加第二届中国品牌商品非洲展等16个国外重要展会及广交会。积极开拓国内市场，举办首届“汕头购物节”、第二届中国（潮南）内衣家居服装节、第15届中国澄海国际玩具礼品博览会，组织“汕货全国行”和“汕货网上行”经贸活动，运用电子商务，将参展线下展会和电子商务线上平台推广结合起来，打造永不落幕的汕货展销平台。成功入选国家首批信息消费试点城市，信息消费规模超过200亿元，促进消费保持畅旺，社会消费品零售总额达到1158.92亿元，增长12.5%。推进乡贤反哺工程，国内外潮商积极参与家乡经济和公益事业建设，潮阳公益基金会累计募集资金21.17亿元，潮南募集资金8.76亿元。

　　五是社会民生持续改善。优先保障民生事业公共财政支出，去年全市共支出132亿元，占公共财政预算支出70.5%，高于全省平均水平。城镇居民人均可支配收入22207元，农村居民人均纯收入10097元，分别增长10.9%、11.8%。居民消费价格指数上涨2.5%，物价保持平稳。新增城镇就业6.16万人，城镇登记失业率为2.4%；农村劳动力培训2.53万人，转移就业5.48万人，就业形势保持稳定。全市参加职工医疗保险48.85万人、企业职工养老保险99.01万人，参加城乡居民医疗保险152.42万人、养老保险185.48万人，完成社保卡发卡437.5万张，基本实现社保卡全覆盖。创建教育强市工作顺利推进，新增10个镇（街）通过省督前检查，龙湖区通过省教育强区督导验收和全国义务教育发展基本均衡区督导评估，金平区申报教育强区督导验收。教育质量继续提升，高考重点线以上高分层学生占全省十分之一，中职教育毕业生就业率达98%。进一步加强教师队伍建设，2.4万名山区、边远农村义务教育教师享受岗位津贴。卫生、教育硬件设施不断完善，启动3家区级人民医院、5家镇卫生院、4家社区卫生服务中心改扩建，新增床位163张；新、改、扩建校舍594所，解决学位约1.5万个。打好计生工作翻身仗，人口计生工作取得新成效。文化体育事业加快发展，市博物馆新馆、侨批文物馆正式免费开放，东征军革命史迹陈列馆、崎碌炮台、文光塔成为我市首批国家级重点文物保护单位；深入开展全民健身活动，南滨全民健身广场建成免费开放。购置公交车108辆，公交车拥有量在全省排名第7位，中心城区公交出行分担率近20%。新开通中心城区到潮阳、潮南区线路4条，同城化公交线路16条，新开通中心城区到厦深高铁潮汕站、潮阳站客运专线4条。全面完成上一轮扶贫“双到”任务，脱贫率达100%。出台老年人权益保障条例，制定民办养老机构优惠措施，资助民办养老服务机构发展，探索农村互助养老新模式，城市居家和日间照料覆盖率分别达到68.6%和75.2%；实施70岁以上老人免费、60岁以上老人半票的公交优惠政策。推进法律服务资源向基层倾斜，建立健全基层村居法律援助网络，68%的村居实现“一村居一法律顾问”。实施水价改革，简化中心城区用水分类，实现工商业用水同网同价。一批残疾人弱势群体的基本民生问题得到解决，直接受惠残疾人超过3万人。深入推进殡葬基本公共服务，殡葬普惠面超过240万人，居全省前列。去年确定的十大民生实事完成年度任务。（具体见附件列表）

　　六是抗灾复产取得全面胜利。去年我市接连遭受“8·17”重大洪涝灾害和强台风“天兔”的袭击，受灾范围之广、受灾人口之多前所未有，直接经济损失超过75亿元。面对突如其来的灾情，自强不息的灾区群众守望相助，奋起自救，有效减少了灾害损失；上级领导高度重视，亲赴一线指挥，从人财物各方面全力支持我市抗灾救灾；全市各级党政把群众的生命安全放在第一位，迅速反应，步调一致，调动一切资源和力量，全力组织救灾复产；军警部队官兵视受灾群众为亲人，不顾个人安危，冲锋在前，奋勇救灾；乐善好施的海内外乡亲心系家乡，捐款捐物，倾力帮助灾区群众渡过难关。灾难面前，各方团结一心、众志成城，汇聚成抗灾复产的强大力量，灾区灾痕得以迅速消除，确保了大灾之后无大疫，灾后复产、重建家园迅速启动，抗灾复产取得全面胜利。据不完全统计，筹措拨付各级救灾复产资金4.11亿元，社会各界筹集救灾善款及物资折合人民币约8900万元，修复水毁水利设施718宗（处）、房屋全倒户重建新房209户，100多万受灾群众基本生活得到妥善保障，6000多家受损企业、商铺全面恢复生产经营。

　　七是社会环境平安和谐。全面开展“平安汕头”建设，积极推进“平安细胞工程”，对“社会治安重点地区”、“社会管理薄弱地区”、“社会矛盾突出地区”三类重点地区实行分类挂牌整治，重拳打击路面盗抢犯罪。着力加强治安巡防管控，建成治安视频监控点20.7万个，有力震慑犯罪行为，全市刑事和治安警情下降12.8%，常住人口发案率相当于全省平均水平的三分之一；“两抢”警情下降8.9%，破获抢夺案件上升71.3%，群众安全感明显增强。创新城市管理体制机制，构建网格化、数字化管理模式，推动管理重心下移和作业层面市场化，启动小贩疏导工程，开展市容环境百日整治行动，集中整治城市管理乱象，市容市貌逐步改善。加强道路交通安全管理，出台实施道路交通安全管理条例、出租汽车客运条例、公共汽车管理条例，加快智能交通系统建设，强化路面监管和现场指挥疏导，交通秩序有所改观。落实“一岗双责”，狠抓安全生产隐患排查治理和“打非治违”，未发生较大以上生产安全事故。

　　八是政务环境明显改善。注重运用特区立法权，提请市人大常委会审议法规议案6件，制定政府规章5件，通过立法推动改革创新。充分发挥法律咨询专家委员会的参谋作用，出台重大行政决策量化标准规定，促进行政决策科学化、民主化、法治化。颁布实施行政复议条例，推动行政复议工作创新发展。深化行政审批制度改革，取消、转移、下放、前移政府职能事项412项，调整比例63.3%。整合政府采购、土地使用权出让、产权交易、工程建设招投标等平台，成立公共资源交易中心。加快电子政务建设，全面启动“汕头政府在线”项目，完成59家单位办公自动化系统建设，42个市级单位555项行政审批和便民服务事项梳理上网，各区县网上办事分厅上线运行，省、市、区（县）三级网上办事大厅互联互通、信息共享，实现政务办公、行政审批、公众服务、效能监察的全流程电子化。整合建设市政府12345统一话务热线，打造集办事咨询、社会救助、投诉举报、意见建议为一体的综合性政府语音服务平台。加强行政监察和审计监督，大力整治公车私用、违规修建楼堂馆所、设立“小金库”和违规使用专项资金等行为，压减公用经费5%。认真贯彻落实中央“八项规定”，深入开展“四治”专项整治行动，大力精减文山会海，市政府全市性会议减少11.7%，以市政府及办公室名义下发文件减少23.3%，政府作风明显改进、行政效能明显提升，创业投资“洼地效应”日益凸现。

　　各位代表！过去一年我们所取得的成绩和进步，是省委、省政府和市委正确领导的结果，是全市人民同心协力、共同奋斗的结果，是海内外乡亲大力支持、积极参与的结果。在此，我代表市人民政府，向奋斗在各条战线的全市广大干部群众、外地来汕建设者，向给予我们支持的人大代表、政协委员、各民主党派、工商联、社会各界人士和海内外乡亲，向中央和省驻汕单位，向驻汕解放军、武警官兵，向所有关心和支持汕头发展的港澳台同胞、海外侨胞和国际友人表示崇高的敬意和衷心的感谢！

　　我们工作中还存在许多不足，经济社会发展还有很多亟待解决的矛盾和问题：一是产业能级不高，传统产业大而不强，新兴产业堆头不大，资源环境制约日益突出，支撑经济持续较快增长的动力和后劲不足，加快发展和转型升级的双重任务很重；二是社会建设特别是教育、医疗、住房、社会保障等公共事业发展相对滞后，底线民生保障不到位，与人民群众的需求差距仍然很大；三是城市管理的科学性、精细化水平亟须提高，交通秩序、市容市貌、生态、治安环境还有不少亟待解决的问题；四是政府机关庸懒散奢作风不同程度存在，一些机关工作人员的时代创新意识和服务理念滞后，行政效能需进一步提升。我们必须正视自身工作中存在的问题，采取有效措施认真解决。

　　二、2014年工作安排

　　新的一年，我们将认真贯彻落实党的十八届三中全会、省委十一届三次全会精神，按照市委十届四次全会的部署要求，牢固树立发展为先、生态为重、民生为本的理念，以全面深化改革为动力，以加快新型工业化和城镇化为突破口，以交通基础设施建设、产业转型升级、中心城区扩容提质为抓手，加快转变经济发展方式，加强社会管理创新，切实保障和改善民生，推动经济社会发展迈上新台阶。

　　今年经济社会发展的主要预期目标是：地区生产总值增长10%，人均生产总值增长9.3%，公共财政预算收入增长10%，固定资产投资额增长25%，城镇居民人均可支配收入增长10%，农村居民人均纯收入增长10.5%，城镇登记失业率控制在3%以内，居民消费价格指数涨幅控制在3.5%以内，节能减排完成省下达年度计划。重点抓好七方面工作：

　　（一）持之以恒开展交通基础设施建设大会战，加快提升区域中心城市便捷通达能力

　　加快打造区域中心城市，外通内联、便捷通达的交通是基础。要紧紧抓住省委、省政府促进粤东西北地区振兴发展，加快交通基础设施建设的契机，持之以恒开展交通基础设施建设大会战，加快提升区域中心城市的对接和辐射能力，破解汕头发展困境。

　　高速公路方面。汕揭梅高速公路汕头段全线建成通车。汕湛、潮惠、揭惠三条高速公路完成征拆工作，实现全面施工。加快潮汕环线高速公路前期工作，着手规划牛田洋快速通道。

　　铁路方面。协调推进厦深铁路通车后相关配套设施的优化和完善。全力推进厦深铁路联络线建设，积极协助、配合完成项目前期工作，争取尽快开工建设。加快汕头港疏港铁路项目前期工作，积极配合省加快完成粤东地区城际轨道规划，争取完成市域轨道交通网规划及报批工作。

　　路桥建设方面。加快国道206线大学路、324线潮阳潮南段和省道235司神线、231凤湾线、237线和337线和惠公路二期工程，以及磊广路、河浦大道、河中路、达南路等改造工程的建设进度。新开工国道324线澄海盐鸿至莲阳段、潮南洋汾陈至石桥头段，省道234线潮阳北闸至河溪段、336线南澳环岛公路等路面改造工程。加快国道324线礐石至红旗岭段、省道335线澄海段、237线潮阳和平至谷饶段等改造工程项目前期工作。南澳大桥建成通车。汕头海湾隧道完成设计并动工建设。完成100公里农村公路建设任务，继续推动危桥整治改造。

　　港口方面。完成马山港区华能汕头电厂重件码头改造工程，力争完成广澳港区防波堤项目50%以上工程量，完成华能海门煤炭中转基地主体工程。新开工广澳港区2万吨级石化码头，争取开工建设两个10万吨级集装箱码头和航道二期工程。积极理顺各种关系，争取尽快重启潮阳港运营。

　　（二）持之以恒推动产业转型升级，加快构建粤东现代产业发展高地

　　加快打造区域中心城市，抢占现代产业发展高地、提升区域综合服务能力是关键。要充分发挥民资民力、侨资侨力的优势，全面实施四大产业计划和“五个100工程”，坚持高端发展取向，推动产业转型升级，引领区域产业发展。

　　大力培育战略性新兴产业。重点做强做大锆产业、北斗导航和卫星遥感产业、大数据产业、环境友好型新材料产业等4个产业。锆产业方面，编制产业规划，出台优惠扶持政策，大力引进上下游企业进驻中国锆城，形成以核级锆材料为龙头的锆系列产品研发、生产、销售、服务产业链条。北斗导航和卫星遥感产业方面，积极开展信息消费试点城市建设，突出行业特色，重点推广面向传统优势产业转型升级的应用。大数据产业方面，依托国际海缆在汕头登陆的优势，积极推进与行业龙头企业开展实质性合作，开展外向型数据业务和智慧能源岛等项目建设。环境友好型新材料产业方面，落实省市共建汕头可降解环保材料产业框架协议，加快基地规划和基础设施建设。积极发展高端游艇、通用航空产业。积极参与“21世纪海上丝绸之路”建设，促进海洋生物医药、海洋高新材料等产业发展。

　　加快发展现代服务业。优先发展金融业，积极引进海外华侨华人和外资金融机构，提高金融机构集聚度和区域服务水平。深化农村信用社改革，推进小额贷款公司和融资担保机构稳健发展。鼓励民间资本发起或参与设立民营银行、村镇银行。推动汕头金融超市建设成为社会金融服务平台，发展第三方支付。重点发展商贸物流业，加快推进苏宁电器广场、龙湖乐园等商业综合体，粤东物流新城、宝奥物流城、广澳物流园等现代物流园区，汇盈、柏亚、五维、宇博等电子商务产业园，粤东（国际）电子交易中心、海吉星华南食品城、国瑞会展酒店等一批专业市场、会展中心建设，推动形成粤东最大的人流、物流、信息流汇聚地。大力发展现代旅游业，重点推进南澳滨海旅游产业园区、潮人码头、潮商旅游休闲游艇码头、海门渔乡风情小镇等项目规划建设。培育发展影视制作、演艺娱乐、文化会展、动漫游戏等文化创意产业。加快发展教育服务业，重点推动高等教育、职业教育扩张性发展。支持汕头大学、粤东高级技工学校办学，办好汕头职业技术学院，全力推动广东以色列理工学院创建工作，推进市高级技工学校新校区等一批职业教育学校建设，为粤东地区乃至全省产业发展提供技术和人才支持。加快发展医疗服务业，科学规划医疗机构布局，推进汕头大学医学院附属肿瘤医院重建、市第二人民医院改扩建、白求恩潮阳医院（养老中心）建设；积极引导社会力量合作办医，启动建设东区2家大型综合医院，策划建设妇幼儿童或牙科等2至3家三甲专科医院，增强粤东医疗服务中心的辐射力。

　　引导扶持传统优势产业高端化发展。抓住入选国家信息消费试点城市的契机，以信息化、品牌等手段和电子商务等现代业态，提升、改良传统优势产业，促进传统优势产业做精做专、做强做大、提质增效。加快建设提升国家日用精细化工产品质量监督检验中心、国家玩具检测重点实验室、国家纺织品安全检测重点实验室、轻工装备研究院、潮南纺织创新基地、专利技术交易与综合利用等一批公共技术服务平台，为传统产业改造提升提供服务。创造政产学研合作发展条件，促进政府、企业、高等院校、科研机构建立技术联盟、产业联盟和标准联盟，推进科研成果产业化。

　　促进园区扩能增效。出台实施全市工业园区建设方案，重新调整园区产业发展布局，明确各园区主导产业，形成一批特色产业园区。整合提升现有园区，推进园区二次开发，盘活存量建设用地，提高土地利用强度和投入产出比。制定出台鼓励支持建设通用厂房、社会组织参与工业园区建设管理等扶持政策，激活民间资本，探索园区市场化运作新模式。加强园区招商引资，运用市场机制和激励机制，依托产业链引进带动力强的好项目、大项目。充分发挥高新区在人才聚集、科技服务、信息服务上的优势，重点扶持孵化一批科技创新型项目和企业增资扩产，发挥效益。积极申报保税物流中心（B型），加速推进保税区升级转型为综合保税区，发挥政策功能优势，以新办高端企业和大宗商品交易市场为载体，实现内外联动、区港联动，促进区港一体化发展。加快建设一批设施农业，畜禽生态健康养殖、休闲生态农业、农产品物流等现代化园区。

　　（三）持之以恒推进城市扩容提质，加快提高区域中心城市宜居宜业水平

　　加快打造区域中心城市，提高城市宜居宜业水平是核心。要持之以恒推进城市扩容提质，打造绿色、低碳、智慧、可持续和宜居宜业的城市，努力提升汕头作为区域中心城市的吸引力和承载力。

　　加快海湾新区建设。进一步梳理海湾新区及华侨经济文化合作试验区的功能定位、实施路径，争取上升为国家战略，探索建立国际化、法治化商事规则，打造世界潮人经济门户、华侨经济文化合作平台和经济特区的升级版。推进海湾新区各功能组团建设，全面完成东海岸新城水利工程建设和验收，中山东路东延段、滨江大道建成投入使用，加快滨海大道、新津河大桥、外砂河大桥及其它市政配套建设，加大招商引资和土地营销策划力度，组织实施新津片区土地出让，深化离岸人工岛项目前期论证；加快推进濠江新城南滨片区土地开发和市政基础设施，以及中轴线景观工程、潮汕历史文化博览中心建设；全面铺开珠港新城土地一级开发，启动市政基础设施和总部经济园区建设；加快粤东物流新城北片区征地，推进泰山路桥项目建设，尽快实现高端物流总部企业集聚。

　　推动城乡综合运营开发。创新城乡发展投融资机制，做大做强城市建设投融资平台，充分利用股权投资基金、债权投资计划、政府债券等各种金融杠杆和市场融资功能，撬动和推进一批重大基础设施和产业项目建设。健全土地储备经营机制，加大中心城区闲置低效土地整合收储力度。结合节约集约用地及城乡土地生态利用制度综合改革试点工作，建立闲置低效用地处置、批而未供土地盘活倒逼机制。深化战略合作，启动西部生态智慧新城土地一级开发和金凤西路、金砂西路、澄海路及大学路立交等路网基础设施建设，推进潮阳东部新城、潮南行政新区、厦深高铁潮阳站（谷饶）物流新城等一批项目开发建设。

　　着力改善城乡人居环境。继续推动城市更新，加快永平路、新兴路、跃进路、外马路、中山中路、中山西路、长平路、金砂路、金环南路、长江路、犁头标路、安居路等一批破损道路、“水浸街”改造。保护提升老城区，保育活化开埠历史文化街区，重点推进永平路100米沿街范围内房产收购、改造。依托绿满家园全民行动、千村环境卫生整治工程，启动“美丽乡村”、“幸福村居”和名镇名村创建活动，在全市农村开展以硬化、亮化、绿化、净化、美化为重点的环境综合整治。大力加强环保基础设施建设，加快推进雷打石环保电厂等一批生活垃圾处理项目，谷饶、贵屿等一批污水处理厂及配套管网建设，启动新溪污水处理厂、龙珠水质净化厂二期工程建设，提高城乡生活垃圾无害化处理率及污水处理率，严控生活污染。加大城乡环境保护和监控执法力度，加强空气质量监测，大力治理工业废气、汽车尾气排放，保护汕头的蓝天白云、清洁空气。

　　提升城乡防灾减灾能力。加强水利基础设施建设，重点开展“百河千沟万渠大整治”行动，用5年时间对全市水利渠系进行大清淤、大清通、大整治，突出解决河道清障和堤防加固问题。提高设防和排涝标准，推进“千里海堤”、病险水库、水闸除险加固和农村重点易涝区整治。按“百年一遇”标准重新规划建设中心城区防洪排涝设施，加固、整修海滨路海堤缺口，完成汕头大围等一批重点水利工程建设。加强防汛抗旱物资储备和应急抢险能力建设，在中心城区、潮南区各建一座市级防汛抗旱物资中心仓库和次中心仓库，各区县各建一座防汛抗旱物资仓库，各镇（街道）、村（居）按照高标准在江河两岸沿线设置防汛砂石仓库和物资堆放点，配齐配足储备物资。加强应急、“三防”指挥系统建设，视频会商延伸到镇，健全完善各级突发事件预警信息发布机构。

　　启动练江流域综合整治。抓住省委、省政府决定综合整治练江流域的契机，按照“五十年一遇”以上标准，加紧做好练江流域综合整治规划编制工作，启动练江潮阳北港、前溪、后溪水闸和潮南新坛、锣鼓陂水闸重建工程，除险加固潮阳海门堤围和潮南练江（井都-成田段）堤围，综合整治潮南南山截流河（陇天-成田段）工程等水利设施配套，逐步从根本上解决练江流域水患问题。严厉整治流域内工业污染源，依法关停、取缔沿岸违规设立的电子拆解、塑料加工、印染、造纸等重污染企业和落后产能，打击电子废物非法入口、露天焚烧、非法酸洗等违法行为，推行深度治理和清洁生产，确保污染物达标排放，力争用5年时间颠覆式改变练江流域整体面貌，改善水体水质。加快贵屿循环经济产业园内集中拆解通用厂房建设，完善园区工业污水处理厂、危险废物转运站及其它环保配套设施，确保拆解户按计划分期分批入园经营，实现集中拆解、集中治污。启动贵屿土壤环境修复工程和北港河底泥修复示范工程。规划建设印染环保工业园，引导印染企业入园集中生产、集中治污。

　　加强城市综合管理。强化市容环境秩序综合治理，落实网格化执法和道路巡回保洁制度，推进小贩疏导工程、集贸市场升级改造工程，整治城市管理“顽疾”。按照“管养分离，重心下移”的要求，加快推动环卫、园林绿化、市政设施维护等作业层面市场化、监督社会化，提高城市管理效益。实施道路、街区形象提升工程，从今年开始，在中心城区开展示范路、示范街创建活动，逐步改善市容市貌。实施道路交通安全条例，坚决取缔报废、无牌无证摩托车、三轮车，实行电动自行车登记注册管理，规范出租车营运秩序，加强中心城区道路临时停车泊位管理；加快智能交通建设，完善交通微循环系统，力争今年道路交通信号灯覆盖率达到56%，确保道路交通安全、有序、畅通。坚持公交优先发展战略，增加投放100辆以上公交车，调整优化公交线网布局，拓展公交服务覆盖范围，增设通往厦深高铁潮汕站、潮阳站等公交驳接线路，提升城市公交服务水平。

　　（四）持之以恒推进经济体制改革，加快破解制约经济发展的体制机制障碍

　　积极稳妥推进市场化改革，发挥市场在资源配置中的决定性作用，充分激发潮商民资和市场活力，增强经济发展的内生动力。

　　全面推进商事制度改革。实施商事主体资格与经营资格相分离，实行商事主体年报备案制度，建立统一的商事主体登记许可及信用信息公示平台。逐步推进“宽进严管”的市场准入体系建设，取消注册资本最低限额，实行注册资本认缴制。大力探索市场准入负面清单管理方式，在海湾新区进行试点，按照“非禁即入”原则吸引各类企业平等进入特许经营领域，争取在现代服务业和基础设施建设等领域有新突破。

　　深化投资体制改革。简化企业投资管理流程，减少政府对企业投资的行政审批事项，对市核准权限内不涉及公共资源开发利用企业投资项目，一律取消核准，改为备案管理，力争实现企业投资审批事项压减50%以上。

　　深化财税体制改革。改进预算管理制度，建立全口径的预算编报体系，强化预算约束力。明确市、区（县）、镇（街道）事权划分，建立各级政府事权与支出责任相适应的体制。完善转移支付体系，压减专项转移支付，提高一般性转移支付占比。积极争取列入地方税综合改革试点。

　　推进金融改革创新。以海湾新区、华侨经济文化合作试验区为载体，规划建设金融商务区，开展金融政策创新试点，争取在人民币跨境双向投融资、人民币资本项目下开放、投资贸易便利化等方面有新突破，吸引金融服务机构集聚。结合电子商务示范城市建设，加快发展新型电子支付业务，争取获得第三方支付牌照。

　　深化国有企业改革。积极发展混合所有制经济，允许非国有资本参股国有资本投资项目。以管资本为主加强国有资产监管，完善国有企业现代企业制度和国有资本经营预算制度。加快市属企业资产重组，突出主业、增强竞争力，打造若干个资产超100亿元的企业集团。

　　着力优化市场发展环境。加快推进社会信用体系建设，编制出台全市社会信用体系建设规划，重点在政务诚信、信用分类监管、农村信用建设等关键领域取得新进展。推进市场监管体系建设，建立全市统一的市场监管信息平台，重点推进市场准入、质量监管、市场交易监管、消费维权、食品安全等五大信息系统，进一步优化市场发展环境。

　　（五）持之以恒加强社会管理和公共服务，加快构筑保障基本民生安全网

　　补齐民生短板，加快解决民生问题，是广大群众的热切期盼、政府工作的重点。要积极回应群众的期待和诉求，持之以恒加强社会建设和管理，推进基本公共服务均等化，着力保基本，构筑起使群众能够安心生产生活的保障网。

　　推动民政工作改革创新。加快民政工作改革创新综合观察点建设，深化社会组织管理改革，建立发展专项基金，鼓励发展行业协会商会类、科技类、公益慈善类和社会服务类社会组织，完善政府向社会组织购买服务制度。推行公益慈善组织社会化运作，培育一批具有潮汕特色慈善组织品牌。探索创新城乡社区服务管理新模式，加快社区公共服务站、儿童友好社区、街道家庭服务中心建设。大力发展社工队伍和志愿服务，培育发展民办社工服务机构，积极开发社会工作岗位，不断拓展社工人才服务领域。推进社会养老服务体系建设，加快市社会福利中心和一批城市居家养老服务示范中心建设，总结推广潮汕特色的农村邻里互助模式，打造集“养老、保健、医疗”于一体的养老模式。提倡移风易俗和严格管理、优质服务并重，巩固和提高殡葬改革成果。深入开展双拥模范城（县）创建和军民共建活动，健全军民融合式发展双拥机制。按期完成村（社区）委员会换届选举工作。

　　加快推进教育创强。做好澄海区溪南镇等13个镇（街道）和澄海区、南澳县申报省教育强镇（街道）、强区（县）工作。推动义务教育均衡发展，提升义务教育标准化学校覆盖率，做好非户籍常住人口子女接受义务教育工作。推进普通高中内涵发展，加快中等职业教育发展，改造薄弱高中学校，全市高中阶段教育毛入学率保持在90%以上。加快发展学前教育，建立0至3岁儿童早期教育服务体系，提高学前教育普惠性，确保85%以上农村乡镇建成普惠性规范化中心幼儿园。重视特殊教育，推进融合教育试点工作。加强教师队伍建设，严格落实义务教育学校绩效工资制度，确保中小学教师工资福利待遇“两相当”。

　　切实改善医疗卫生公共服务。优化医疗资源布局，提高基层医疗卫生服务能力，加快推进3家区级人民医院、4家社区卫生服务中心改扩建。加快基层卫生人才培养力度，公开招考一批医学院毕业生充实到基层医疗机构。扎实推进镇村卫生服务一体化管理，促进基本公共卫生服务重心下沉。巩固完善基本药物制度和基层运行新机制，深入推进公立医院改革，促进基本医疗和公共卫生服务均等化。加强人口计生服务和管理，稳定低生育水平，提高人口素质，促进人口长期均衡发展。

　　加快文化体育事业发展。大力推进基层文化设施全覆盖工程，实现乡镇综合文化站100%达标。全面实施公共文化设施免费开放，推动民办文化场馆向社会开放。建立健全全民健身公共服务体系，完善城乡社区体育健身设施，广泛开展全民健身活动。加快市档案馆新馆建设，积极推进数字图书馆、数字文化馆建设。推进艺术精品创作，提高文化艺术品味，加强文化遗产保护，重点推进潮汕文化生态保护区建设。加强城市文化形象推介，打造城市文化品牌。

　　全力维护社会稳定。深入推进“平安汕头”建设和“平安细胞工程”，夯实平安区县、平安乡镇、平安社区的创建基础。加快社会治安视频监控等防控设施建设，着力打造群防群治的社会治安立体防控体系，完成4000个一类、36000个二类视频监控点建设任务。进一步完善重大决策社会稳定风险评估、维稳和治安形势分析、矛盾纠纷排查化解和应急处置工作机制，开展维稳处突演练，切实维护社会大局稳定。严格落实安全生产“党政同责、一岗双责、齐抓共管”机制，建立健全日常检查监督制度和隐患排查治理长效机制，突出重点行业领域安全监管，坚决遏制较大以上生产安全事故，努力实现安全生产形势持续好转。

　　（六）持之以恒办好十大民生实事，加快解决事关广大群众切身利益的实际问题

　　今年市级财政投入7.16亿元，继续办好十大民生实事。

　　一是提高困难群众生活保障水平。城乡低保月人均补贴水平提高到333元和160元，农村五保供养标准不低于当地2013年度农村居民人均纯收入的70%，孤儿基本生活集中供养和分散供养标准分别提高到每人每月1150元和700元，城镇“三无”人员集中供养和分散供养基本生活费标准每人每月1000元和800元。

　　二是配置乡镇卫生院“五个一”设备。为全市25所一般乡镇卫生院、8所中心卫生院配置救护车、全自动生化仪、心电图仪、X光射线机、B超仪等设备。

　　三是提高基本医疗保障水平。城乡居民基本医疗保险参保补助标准由280元提高到320元，医疗救助对象政策范围内医院自付医疗费用的救助比例提高到70%，启动农村妇女“两癌”（宫颈癌、乳腺癌）免费检查试点，改扩建市第二人民医院住院综合大楼。

　　四是实施教育普惠工程。继续提高义务教育生均公用经费标准，小学从每人每学年750元提高到950元，初中从每人每学年1150元提高到1550元；扩大中等职业教育免学费范围，完善中等职业教育免学费和助学金制度。

　　五是加大残疾人救助力度。启动市慈爱托养中心建设，实现残疾人公立照料服务新突破；市特殊教育学校建成并投入使用，今年9月全面招生办学；提高重度残疾人护理补贴和贫困残疾人生活津贴，重度（二级）残疾人的护理补贴每人每年由600元提高到1200元，贫困残疾人生活津贴由每人每年100元提高到600元；实施贫困精神病患者、白内障复明、抢救性康复训练等残疾人医疗康复救助，为600名长期卧床的特困（低保）残疾人提供一年每人3000元的家居护理或一次性医疗补助。

　　六是实施文化体育惠民工程。完成市文化馆、市非遗馆改造升级和南昌起义南下部队指挥部旧址修缮布展，免费开放；达标升级建设10个综合文化站、300个行政村（社区）文化室；每月每个行政村放映一场公益电影，每月组织两次潮剧潮艺进社区、农村、企业演出；实行百所中小学校体育设施向社会开放；建设一批社区体育公园。

　　七是继续实施“绿满家园”全民行动。加快推进西堤公园重建、石炮台公园等绿地改造，实施道路绿化“全覆盖”工程，启动一批森林生态项目建设，打造一批绿化美化示范村。

　　八是继续推进千村环境卫生整治。在继续对全市农村生活垃圾、生活污水进行全面整治的同时，重点推进雷打石生活垃圾卫生填埋场扩建工程，新建潮阳、潮南垃圾焚烧发电厂。

　　九是继续推进城镇保障性住房建设。筹集公租房1240套，发放租赁住房补贴1100户，基本建成3100套。

　　十是实施用水治水工程。继续实施潮阳、潮南村村通自来水工程，启动练江流域综合整治水利设施配套建设，开展“百河千沟万渠大整治”行动。

　　（七）持之以恒打造阳光法治服务政府，加快优化创新型经济特区政务环境

　　打造行为规范、运转协调、公正透明、廉洁高效的政务环境，是再创特区发展新优势的迫切需要，必须进一步改进行政管理方式，深化行政审批制度改革，持之以恒推进阳光法治服务政府建设。

　　着力加强科学民主依法决策。自觉接受市人大及其常委会的法律监督、工作监督和政协的民主监督，自觉接受群众监督和舆论监督。坚持公众参与、专家论证、风险评估和集体决策机制，落实重大决策合法性审查制度，确保民主决策的科学性和准确性。加强对行政决策的全过程监督，组织开展全市依法行政考评活动，建立健全监察、审计部门负责人列席政府常务会议制度。

　　深化行政管理体制改革。加快推动政府职能转变，完成市县两级政府机构改革，全面梳理部门行政职权，出台政府部门权力清单。推进行政审批法定化、标准化建设，再造和优化审批流程。积极探索相对集中的行政审批权制度改革，推进行政审批“三集中”工作，实现政府部门的审批职能、机构、权限向窗口集中，逐步建立“审、管”分离、“监、管”分离运行机制，促进审批提速增效。加快推进“汕头政府在线”项目，完成系统建设工作，构筑全市统一的电子政务应用平台。完善市网上办事大厅和区县网上办事分厅建设，促进行政服务中心的实体窗口和网上办事大厅的虚拟窗口有效衔接。

　　着力加强廉政建设。强化政府机关工作人员廉洁从政教育和廉政文化教育。健全规范权力、防止利益冲突等制度，党政正职不直接分管人事财务等重要部门工作，推进廉政风险控制。深入开展党的群众路线教育实践活动，认真落实中央“八项规定”精神，坚持不懈纠正“四风”。深入实施预防腐败条例，推进政府系统惩防体系和制度建设，重点加强领导干部和国有企业领导人员经济责任审计，强化效能监察、执法监察和廉政监察，严肃查办庸懒散奢等行为，促进干部清正、政府清廉、政治清明。

　　各位代表，今年经济社会发展任务艰巨，责任重大。让我们在省委、省政府和市委的坚强领导下，紧紧依靠全市人民，凝聚海内外潮人的智慧和力量，攻坚克难，扎实工作，努力开创特区经济社会振兴发展、跨越发展的崭新局面！
